# Sustainable Grassland-Management Systems and Their Effects on the Physicochemical Properties of Soil

**DOI:** 10.3390/plants13060838

**Published:** 2024-03-14

**Authors:** Urška Lisec, Maja Prevolnik Povše, Anastazija Gselman, Branko Kramberger

**Affiliations:** 1 Department of Chemistry, Agrochemistry and Pedology, Faculty of Agriculture and Life Sciences, University of Maribor, Pivola 10, 2311 Hoče, Slovenia; 2 Department of Animal Science, Faculty of Agriculture and Life Sciences, University of Maribor, Pivola 10, 2311 Hoče, Slovenia; maja.prevolnik@um.si; 3 Department of Grassland and Fodder Production, Faculty of Agriculture and Life Sciences, University of Maribor, Pivola 10, 2311 Hoče, Slovenia; anastazija.gselman@um.si (A.G.); branko.kramberger@um.si (B.K.)

**Keywords:** grazing, cutting, combined system, soil organic carbon, soil inorganic carbon, grassland long-term management, soil physicochemical property

## Abstract

Grassland covers approximately 17.4% of Europe’s land area, stores about 20% of the world’s soil carbon and has the potential to sequester carbon. With the help of sustainable management systems, grasslands could reduce greenhouse gases and act as a terrestrial sink for atmospheric CO_2_. In this study, we will investigate the effect of grassland management (cutting, grazing, and a combination of the two) and soil depth (0–10, 10–20, 20–30 cm) on the physical (volumetric water content—VWC, bulk density—BD, porosity—POR, mass consisting of coarse fragments—FC) and chemical properties of soil (organic carbon—SOC, inorganic carbon—SIC, total carbon—STC, total nitrogen—STN, organic matter—SOM, C/N ratio, pH) in Central European lowlands. The management system affected BD, SOC and STN and tended to affect VWC and STC in the first soil depth only. Grazing and the combined system stored greater amounts of STN, SOC and STC and had higher BDs at the surface (0–10 cm) compared to the cutting system. Most soil properties were influenced by soil depth, with C/N ratio and BD increasing and SOC, STC, STN, SOM, VWC and POR decreasing with depth. Our study highlights an opportunity for grassland users to improve soil quality, reduce fossil fuel usage and improve animal welfare through their management systems and argues that systems such as grazing and the combined system should be promoted to mitigate climate change.

## 1. Introduction

Grassland is the most effective resource for greenhouse gas mitigation in agriculture [1] and is the most important terrestrial organic carbon pool [2]. Natural and semi-natural grasslands store approximately 30% of soil organic carbon [3] and reduce greenhouse gas emissions produced by the agricultural industry by safely storing atmospheric carbon dioxide (CO_2_) in the soil [4,5,6,7,8]. Improved sustainable management of grassland will promote global responsibility by preserving and restoring vital ecosystems [9,10]. A well-managed grassland ecosystem increases soil organic matter [2,11,12], acting as a net-carbon sink [13,14] and storing twice as much carbon as the entire atmosphere [15,16]. Climate-resilient management of grasslands can maintain biodiversity [4], conserve ecosystems, contribute to food production and influence broader ecological processes [17].

Permanent grasslands cover 40% of the Earth’s land surface [3], comprise 70% of agricultural land and store 12.3% of global carbon [18]. They are increasing in Europe, covering about 17.4% of the total land area [19]. Agriculture contributes to anthropogenic greenhouse gas emissions by producing large quantities of N_2_O (78.6%) and CH_4_ (39.1%) [20]. These emissions can be reduced through sustainable grassland management by restoring soil organic carbon (SOC), especially in the top 30 cm [13,21,22] of the soil, where 80% of the root biomass is located [9]. It has been determined that 1 Mg of SOC removes as much as 3.67 Mg of CO_2_ from the atmosphere [18]. Improved management strategies can increase soil carbon by 0.105 to over 1 Mg C ha^−1^ per year, with an average of 0.47 Mg C ha^−1^ per year^−1^ [23]. This increases soil fertility and reduces the need for synthetic nitrogen fertilizers, which in turn lowers N_2_O emissions [24]. Higher SOC storage in the soil also reduces CH_4_ emissions by improving soil structure and fertility, promoting deeper-rooted grass species and improving anaerobic conditions [25].

Maintaining soil carbon and nitrogen levels plays a central role in mitigating global warming and ensuring ecological security [15,26]. Nutrient fertilization, livestock grazing, and the liming and cutting frequency of grasslands affect SOC and soil total nitrogen (STN) stock [9,27]. Compared with SOC, soil inorganic carbon (SIC) accounts for one third of global soil carbon and is key to the carbon cycle. SIC plays a crucial role in the global carbon cycle by sequestering carbon from the atmosphere and storing it in the soil as bicarbonate, which contributes significantly to terrestrial carbon stocks [28]. SIC is strongly dependent on geological soil properties [16] and increases linearly with depth [10]. In Europe, the average SIC content is 31 Mg ha^−1^. Despite its importance, SIC is often overshadowed by SOC because it forms gradually from parent material and is exchanged with atmospheric CO_2_ at a slow rate [29]. Therefore, the impact of land-management practices on soil carbon levels needs to be evaluated and better understood.

Grazing and cutting, the most common grassland-management systems, can have different effects on SOC, SIC and STN through direct and indirect pathways [10,30,31]. Different methods of returning biomass to the soil can alter root exudates and affect the formation of SOC and storage in belowground processes [32]. Cutting typically removes plant biomass within a day, leaving up to 20% of the total harvested biomass as green cuttings, which are considered to be crop losses [33]. With cutting, only plant residues are taken up, whereas, with grazing, 50–70% of the removed biomass is later returned to the soil in the form of excreta [3]. The higher net-carbon storage after grazing (141 g·C·m^−2^ year^−1^) compared to cutting (22.7 g C m^−2^ year^−1^) confirms a higher potential to sequester carbon under a grazing system [34]. Therefore, we suggest that the grazing system may offer a greater contribution to carbon sequestration than the cutting system. Many studies have demonstrated that optimizing grazing systems increases soil carbon sequestration [4,23,35,36]. While some researchers have investigated the individual effects of cutting and grazing on SOC, they have seldom been combined. Insufficient research has been conducted regarding the effects of combined management on total organic carbon and nitrogen content distribution and storage. The combination might promote considerable positive feedback for plant production through soil-nutrient cycling [30]. Therefore, to improve grassland ecosystems, it is necessary to determine how combined management practices affect the storage of SOC, SIC and STN at different soil depths. 

Consequently, there is a significant gap in the literature regarding the amount of organic and inorganic carbon in deep soils and their sensitivity to management, especially grazing compared to cutting. Our results will have implications for future grassland-management strategies with regard to carbon storage and global carbon models. This work aims to summarize information on grazing management, cutting and the effects of combining both management types on SOC, SIC and STN content. We hypothesize that grassland management affects soil physicochemical properties at different soil depths. We also hypothesize that grazing and combined systems increase the amount of STC and STN in the first few centimeters of soil. Therefore, a better understanding of land-use practices could provide better guidance for developing appropriate management practices that prevent soil degradation and contribute to carbon sequestration [30]. Our research provides a scientific basis for determining the optimal level of land-use intensity needed to maintain healthy and productive grassland ecosystems in Central Europe.

## 2. Results

### 2.1. Physical Properties of the Soil under Different Grassland-Management Systems

The effects of the management system and soil depth on the physical properties of the soil are shown in Table 1. In general, soil depth significantly affected all physical properties of the soil, while the management system only affected the VWC, and the effect of the management system × soil depth interaction on all physical properties was insignificant. We further analyzed the effect of the management system on these properties within each soil depth and vice versa (Table 2). The results revealed that the management system had a significant impact on the BD and a tendency to affect VWC, though only at a depth of 0–10 cm. In the first soil depth, the BD was significantly higher (*p* = 0.039) in the grazing system (1.12 g cm^−3^) than in the cutting system (1.01 g cm^−3^), with the combined system showing intermediate values (1.09 g cm^−3^). The tendency of the highest concentration of VWC was in the first soil depth observed in the grazing system (47.49%), followed by cutting (43.87%) and the combined system (41.99%). POR and CF were unaffected by grassland management, irrespective of soil depth. Soil depth had a highly significant effect (*p* < 0.001) in all three grassland systems on POR and BD, and mainly on VWC. According to the results, BD increased significantly with depth, while POR and VWC decreased. For CF, soil depth had no effect on any of the management systems.

### 2.2. Chemical Properties of the Soil under Different Grassland-Management Systems

**Figure 1:**
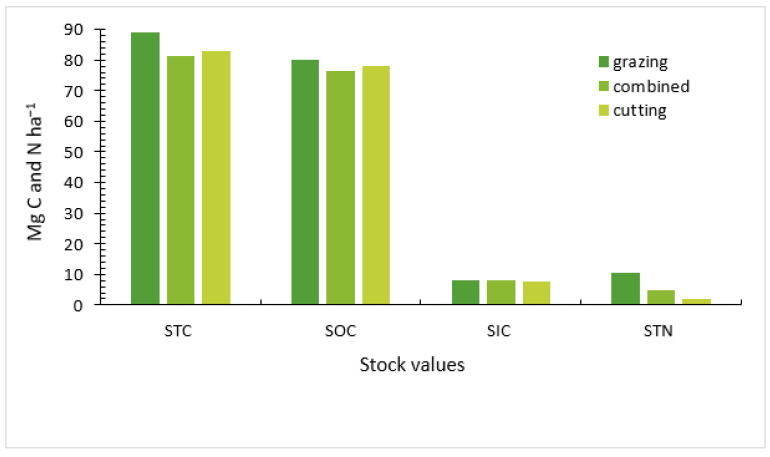
STC_stock_, SOC_stock,_ SIC_stock_ and STN_stock_ in soil under three grassland-management systems (cutting, grazing and combined system) at 30 cm depth. SOC—soil organic carbon; SIC—soil inorganic carbon; STC—soil total carbon; STN—soil total nitrogen.

The effects of the management system and soil depth on the chemical properties of the soil are shown in Table 1. In general, soil depth significantly affected five out of seven chemical properties of soil (SOC, STC, STN, C/N, SOM). The effect of the management system and the effect of the management system × soil depth interaction were insignificant for all chemical properties. Further analysis was conducted to determine the effects of the management system on the chemical properties within each soil depth and vice versa (Table 3 and Figure S1). The results show that the management system has a significant effect on the STN and a tendency to affect the SOC and STC, but only at a depth of 0–10 cm. For the C/N ratio, a significant effect of the management system was observed at a depth of 20–30 cm. In the first soil depth, STN was significantly higher (*p* = 0.039) in the grazing system (3.73 Mg ha^−1^) than in the combined (3.55 Mg ha^−1^) and cutting system (3.21 Mg ha^−1^). The highest SOC was observed in the first soil depth managed using the grazing system (36.34 Mg ha^−1^), followed by the combined (33.62 Mg ha^−1^) and the cutting systems (32.10 Mg ha^−1^). A similar trend was observed for STC at a depth of 0–10 cm, with the highest values in the grazing system (38.35 Mg ha^−1^), followed by the combined (35.55 Mg ha^−1^) and the cutting system (33.59 Mg ha^−1^). For the C/N ratio, the values were significantly higher for the grazing system compared to the combined and cutting systems (16.18, 11.57 and 11.56, respectively) in the third soil depth only. The SIC, SOM and pH were not affected by the grassland management, irrespective of soil depth. Soil depth had a highly significant effect (*p* < 0.001) on SOC, STC, STN and SOM (all of which decreased with depth) in all three management systems and on the C/N ratio in the first depth, showing an increase with the soil depth. For pH and SIC, soil depth had no significant effect for any of the management systems. Additionally, stock values at the depth of 0–30 cm were compared for STCstock, STNstock, SICstock and STNstock (Figure 1). Somewhat higher values for all four chemical properties were observed in the grazing system but did not differ significantly from the other two management systems.

### 2.3. Relationship between the Physical and Chemical Properties

**Figure 2:**
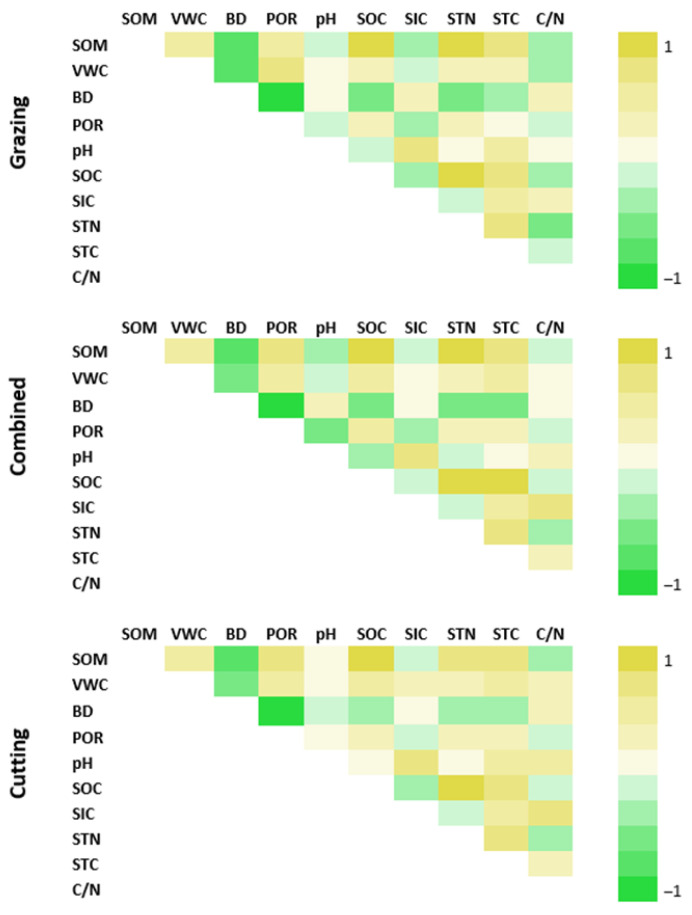
Correlations between physicochemical soil properties for cutting, grazing and combined systems at 0–30 cm. SOC—soil organic carbon; SIC—soil inorganic carbon; STC—soil total carbon; STN—soil total nitrogen; SOM—soil organic matter; VWC—volumetric soil water content; BD—bulk density; C/N—carbon/nitrogen ratio; POR—porosity.

The results of the Pearson correlation analysis are presented in Figure 2. There were strong significant correlations between BD, POR and VWC for all three management systems. WWC was positively correlated with POR (r = 0.43 to 0.75) and negatively correlated with BD (r = −0.74 to −0.73), with the highest correlation coefficient observed for grazing. BD showed strong negative correlation with POR in all three management systems (r = −0.85 to −0.91), while SOM was significantly correlated with STN (r = 0.79 to 0.84, *p* < 0.001) in all three management systems. A significant negative correlation was found between SOC and BD in all management system, with higher values for the grazing and combined systems (r = −0.55 to −0.61, *p* < 0.001) compared to the cutting system (r = −0.34). A significant correlation between SIC and pH was found for the grazing (r = 0.74, *p* < 0.001), combined (r = 0.66, *p* < 0.001) and cutting (r = 0.61, *p* < 0.001) systems. A significant negative correlation between STN and C/N was observed in all three management systems (r = −0.34 to −0.54).

## 3. Discussion

### 3.1. The Effects of Different Types of Grassland Management on the Physical Properties of the Soil

Management systems have different effects on the soil properties, which is particularly evident in the distribution of the stone content at different depths. Deeper layers, from 10 to 30 cm, consistently have a higher proportion of stones (<2 mm). In addition, mineral particles support a stable soil structure and promote the permanent storage of organic carbon. Soil porosity showed a significant correlation with volumetric VWC and SOM across all three management types. This may be because SOC promotes greater aggregation between soil particles, resulting in the formation of stable aggregates, and thus increases soil porosity by increasing water uptake [37]. Our results show that porosity under grazing was greater at the second and third depths than in other systems. 

Soil BD in the topsoil of grassland may be directly related to grassland management, e.g., stocking density and machinery use [36]. Our results revealed that grazing has a direct effect on subsoil BD, as observed in research where grazing intensity increases soil BD [7,27], as intensive management appears to increase BD in the second and third depths. Pastures have the highest BD, as animal/ruminant activity causes high soil compaction at the first depth [7]. Fynn et al. and Sonneveld et al. [36,38] reported that STC is negatively correlated with soil BD, which is in agreement with our results for each grassland-management method.

### 3.2. The Effects of Different Types of Grassland-Management Systems on the Chemical Properties of the Soil

The study investigates the influence of different management systems on the SOC, SIC and STN content across various depths. Specifically, higher levels of SOC, SIC, STC and STN were observed in the first soil layer under grazing, followed by combined and cutting management systems. Our results show that utilizing grazing and combined systems tends to increase the SOC content in the topsoil (0–10 cm) compared to the cutting system. Similarly, the STN concentration in the same depth is significantly higher in the grazing than in the cutting system. These findings are in agreement with several previous studies (e.g., Li et al., Sonneveld et al. [26,36]), which found the highest concentration of SOC and STN in the first depth (0–10 cm) under the grazing system. Recently, Leifeld et al. [2] reported that carbon stocks in the upper depth (0–8 cm) were higher in grazing systems than in cutting systems. 

The higher contents of SOC, SIC and STN in the first depth under the different management systems could be explained by a higher C input in the grazing and combined systems compared to the cutting system. The grazing system results in higher carbon concentrations in the soil than non-grazed systems [39]. A study investigating the influence of long-term grazing on organic and inorganic carbon content found that heavy grazing resulted in a higher total soil carbon content, with 68% of the increase being a result of higher SIC value levels [40]. This implies that ecosystems are more resilient to grazing than they are to cutting [30]. These findings are consistent with published studies [26,41] reporting that the cutting area had a lower litter quality and decomposition rate than the grazed area due to lower water availability and slower nutrient cycling, which affected the SOC stock and STN stock. Unlike grazing, cutting does not lead to the deposition of excrement, which can affect the nutrient cycle in the soil [34,42] and indirectly stimulate root exudation in plants [27,30]. 

The samples were taken from the first 30 cm, as, according to Bajtes [43], on average, 39–70% of the total organic carbon in soil is found in the top 30 cm worldwide, and almost 44% is found in the top 30 cm in Central and Eastern Europe. Studies have shown that SOC decreases more with increasing depth under grazing than under a cutting system [2]. Grazing systems directly promote microbiological products, which can influence the stabilization of (and consequent increase in) soil organic material around soil minerals [27]. The SOC, STC and STN content in our study decreases significantly with increasing soil depth, which is also consistent with the results presented by Li et al. [26], indicating that there is a risk of large amounts of SOC and TSN being mineralized and transported out of the surface soil by erosion processes. Frequent grazing may lead to higher soil deposition than cutting or a combined system. The higher carbon content in the first depth of the sampled soils could be due to the higher carbon input from grazing compared to cutting, with recycling accounting for about 50–80% of plant biomass, which could influence the higher SOC and STN content under grazing [32,44]. According to Chen et al. [3], the order of the SOC and STN concentration decreased as follows: grazing < combined management < control. The findings of Leifeld et al. [2] were similar to ours, but without a cutting system, they found that the SOC content was significantly higher in the grazing system. They suggested that this was due to the incorporation of plant material as a result of trampling during grazing. The higher SOC content could also have been a result of the livestock population and the deposition of excreta, which stimulates the flow of nutrients back into the soil and subsequently increases the nutrient content [45]. 

In their study, Chen et al. [3] show that grazing twice a year and grazing combined with cutting improves carbon and nitrogen sequestration in the soil by increasing the productivity of the grazed areas. The latter is also consistent with studies in which, in a multi-year experiment, the amount of aboveground biomass was significantly higher following combined utilization than after grazing and control. Combined management can increase SOC or STN, but it is less common in Slovenia compared to the other two practices. Cutting leads to rapid changes in the amount of N and C stored in the soil through compensatory growth, while long-term grazing can reduce the number of plants that fix N [46]. Although the residual dung left behind after grazing can increase the mass of microorganisms, heavy grazing leads to a greater depletion of nitrogen in soil [47]. In our study, this could lead to a higher STN at a depth of 10–30 cm in the cutting system. Therefore, the lower pH value leads to a low decomposition rate, and microbial respiration is strongly reduced compared to less acidic soils [48]. 

A significantly strong correlation between SOC and STN was found for all types of grassland-management systems, contributing to an increased water-holding capacity, improved soil structure, and increased biological activity [49]. This result is consistent with another study [46], in which the increase in SOC content was strongly correlated with high STN content. Our results are in agreement with the study of Pringle et al. [50], who also found an association of SOC with STN and SOM. In their work, a significant strong correlation was found between STN and SOM in all types of grassland management, with the highest one in the combined system, which maintains plant species diversity; this is crucial for the stability of the ecosystem and the optimization of soil structure by reducing cattle grazing [47]. Our results not only confirm this explanation, but also show that grazing has the opposite effect on the volumetric water content (VWC) of the soil in all soil depths. In addition, the findings of Jafarin et al. and Li. et al. [13,26] support the notion that different soil properties resulting from different land uses in the topsoil can influence soil organic carbon (SOC) and total nitrogen (STN). This is consistent with the results of [16], in which 70 Mg of SOC ha^−1^ was found in the top 30 cm of the pasture. The considerable input of plant litter and residues provides a plausible explanation for the increased carbon levels observed in the grazing system.

Organic matter production and decomposition control SOC content, as do temperature and precipitation, which are the most important factors in soil organic matter dynamics [9]. A higher C/N ratio in the second and third depth indicates a lower decomposition rate of organic matter during grazing, which is an additional factor that allows more STC and STN to be obtained during grazing. The SOC content has a major influence on the physical structure of the soil and various ecosystem services (e.g., water retention) [7]. Soils with a higher organic carbon content tend to store more water [51]. This was also confirmed by our research, as the first two depths at which STC is highest during grazing also have the highest proportion of VWC. The strong correlation between SOM and VWC in each management system confirms that SOM increases the available water capacity. Each percent of organic matter increases the available water capacity by approximately 1.5% [13], as higher SOM improves water infiltration and the water-storage capacity of the soil [38]. These results were confirmed by recent studies conducted at the same site, where the grassland maintains water and climate regulation [17]. Our results also confirm the correlation between SOM and water infiltration, as the SOM is at its highest with grazing at 0–20 cm depth, as is the average volumetric water content (VWC) of the soil. The effects of pasture management on SOC are influenced by soil cover and a high organic matter supply [7]. Fynn et al. [38] reported that SOM content favors aeration and the reduction of bulk density by increasing soil porosity, which was also confirmed by our results. The lowest C/N ratio was found in the first two depths (0–20 cm) of the combination, indicating a better availability of nitrogen and a faster decomposition of organic matter. The latter is also supported by our results, as the lowest C/N ratio of the SOM was found following the combination treatments in the first two depths.

In all three management systems, a lower pH was observed at the surface (0–10 cm) compared to the deeper depths, which is consistent with a previous study by Gilmullina et al. [32]. Grazing systems had the highest pH at all three depths, mainly due to the inorganic carbon content. We found that the cutting system with the lowest pH at the second and third depths had higher SOC concentrations than the other two systems, what is in accordance with study showing a significant trend of increasing SOC concentration with increasing soil acidity [52]. This is consistent with studies showing low soil pH reduced microbial biomass and activity [52,53]. 

The SIC content of soil is influenced by both the parent material and the climate. Generally, the SOC content is greater in the upper soil depth, whereas the SIC content is lower in surface horizons and higher at deeper soil depths [10,16]. The highest SOC content was observed up to a depth of 40 cm [29]. Carbon inputs from aboveground biomass, litter and roots are major contributors to the storage of organic carbon in surface soils. Liu et al. [54] found a decrease in soil inorganic carbon (SIC) during the grasslands restoration. This was mainly due to a reduction in soil pH connected to an increase in CO_2_ concentration and soil water content, which favored the dissolution of SIC. In our research, the management system and soil depth did not have a statistically significant effect on the amount of SIC, but several previous studies have found that SIC is concentrated in deeper soil horizons of 30–70 cm [29]. These results are consistent with Chang et al. [8], who also found a significantly higher SIC content below 20 cm depth. Grazing can lead to higher soil carbon concentrations than those found in non-grazed systems [55,56]. A study investigating the impact of long-term grazing on organic and inorganic carbon content found that heavy grazing led to higher total soil C content, with 68% of the increase resulting from higher SIC [55]. Based on long-term studies [39], it can be assumed that the average annual increase in SOC content was 0.13 Mg C ha^−1^ under heavy grazing compared to ungrazed grassland. In the case of SIC, the increase was 0.29 Mg C ha^−1^ y^−1^ at a depth 0–90 cm. 

Our results can provide a scientific basis for policy decisions regarding how land use should be approached to preserve soil functions and, thus, productive grassland. Considering all the advantages and disadvantages of grazing and cutting, we suggest that grazing in combination with cutting could be a suitable sustainable management system for semi-natural grasslands, which could promote significant positive feedback on crop production through the intermediate nutrient cycling in the soil. However, the combined effects of grazing and cutting on soil nutrients and soil-nutrient cycling are in need of further investigation. Research shows that grazing provides better ecological outcomes for carbon and nitrogen storage in terms of maintaining soil processes in semi-natural lowland grasslands in Central Europe, which is consistent with previous studies by Gilmullina et al. [32]. In general, the effects of cutting in combination with grazing on soil properties were similar to those of grazing alone, suggesting that cutting already-grazed areas does not have additional negative effects on soil properties. Thus, we surmise that a combination of cutting and grazing could increase the sustainability of soil processes, especially at 10 cm depth.

## 4. Materials and Methods

### 4.1. Site Description

The study was conducted in the eastern part of Slovenia (from 46°33′07.5″ N 14°53′50.3″ E to 46°28′26.7″ N 15°59′58.9″ E) in an area where 72% of all livestock farms are located. Prior to the sampling in 2020, the owner of the farm was interviewed on-site to obtain a detailed history of the area. The selected dairy farms with permanent grasslands have been under the same management for more than 30 years. The participating farms were chosen for their pedoclimatic characteristics; in particular, the Eutric Cambisols soil type (textural classes silty clay loam, silty loam, and silty clay [57]) and an altitude 200 to 400 m above sea level. During the growing season from April to October 2020, the mean annual air temperature was 10.3 °C and the mean annual precipitation was 789 mm. The study included three different grassland-management systems: cutting (3 or 4 cuts per year), grazing (the stocking rate was 1.6 livestock units per hectare), and a combined system (combination of 2 or 3 cuts in summer and grazing in autumn). The fertilization of the grassland systems depended on the management system; in the cutting system, slurry (10 m^3^ ha^−1^) was applied to the cut areas after each cut. For the combined management system, slurry (15 m^3^ ha^−1^) was applied after each cut except the last. No additional fertilizer was applied to the areas where the dairy cows grazed daily from mid-April to mid-October. In all three systems, mineral fertilizers containing phosphorus were only applied in spring during the first fertilization. In the systems where cutting was included, 40 kg P_2_O_5_ ha^−1^ per year was generally applied, as was 70 kg P_2_O_5_ ha^−1^ per year in the grazing system. All measurements were taken in autumn 2020, after the end of the growing season. 

### 4.2. Field Sampling and Measurement of Soil Sample

Soil samples were collected from 72 farms using three grassland-management systems based on the actual frequency of grassland use in the region: cutting (40 farms), grazing (20 farms) and combined systems (12 farms). The samples were obtained from 72 locations with three replicates for physical properties and ten replicates for chemical properties. Soil samples obtained to determine the SOC, SIC, STN, SOM and pH were collected at a depth of 30 cm separately for each depth (first depth: 0–10 cm, second depth: 10–20 and third depth: 20–30 cm) using a thin auger (1 cm diameter). Ten randomly distributed soil samples were collected from each plot.

### 4.3. Sampling and Measurement of Soil Physical Properties

Samples for the determination of soil bulk density (BD) were collected at each depth in three replicates (first, second and third depth) using the core method. The determination was performed by taking undisturbed cylindrical soil cores 7 cm in diameter and 10 cm high, which were weighed before and after drying at 105 °C. The average bulk density of each replicate was reported and used to calculate the SOC, SIC and STN stock. Soil porosity (POR) was obtained from known BD and soil particle density (2.65 g cm^−3^) [58]. The determination of the volumetric water content (VWC) was also based on the latter method with the additional determination and calculation of the gravimetric water content (W). The samples taken to determine the chemical properties of the soil were air-dried, ground and sieved to 2 mm. The residue left on the 2 mm sieve was a mass consisting of coarse fragments (CF). 

### 4.4. Determination of Soil Chemical Soil Properties

The soil samples were analyzed at the Alice Holt Research Station, Forest Research, UK. All visible plant material and stones were removed from the samples prior to analysis. The subsamples were then air-dried, ground, sieved to 2 mm, ball-milled, homogenized to mg level and analyzed to determine their total carbon (TC) and total nitrogen (TN) content according to the international standards ISO 10694 [59] and ISO 13878 [60]. The measurements were obtained using a Carlo Erba CN Flash EA1112 analyzer (CE Instruments Ltd., Wigan, UK). First, 30 mg of soil was weighed and placed in a tin capsule. The subsample was then burned in a furnace at 900 °C under an influx of oxygen and in the presence of an oxidation catalyst. The C and N species were separated using a gas chromatography column and detected using a thermal conductivity detector. To determine the amount of soil inorganic carbon (TIC), the subsamples were placed in an oven at 500 °C for 2 h to remove the organic carbon. Finally, the amount of organic carbon in the soil (C_org_) was indicated by the difference between the TC and TIC measurements.

The pH value was determined according to the international standard ISO 10390 [61], which specifies an instrumental method for the routine determination of the pH value with a glass electrode in a 1:5 (volume fraction) suspension of soil in water.

### 4.5. Data Calculation

The volumetric water content (VWC) was calculated for each depth (i) according to the following equation:

VWC_i_ (%) = W_i_ × BD_i_ (1)

The soil organic carbon content (SOC) was calculated for each depth (i) according to the following equation [62]:

SOC_i_ (Mg C ha^−1^) = Corg_i_ × BD_i_ × T_i_ × (1 − CF_i_), (2)

where

C_i_ = the proportion of organic carbon mass in the depth increment i.

BD_i_ = the bulk density in g cm^−3^ at a depth of i.

W_i_ = the gravimetric water content at a depth of i.

T_i_ = the thickness of the soil depth (cm) at a depth of i.

CF_i_ = is the fraction of the mass consisting of coarse fragments > 2 mm in diameter at a depth of i. 

The SOC was recalculated for each depth layer from 0 to 30 cm. Total soil nitrogen (STN) and soil inorganic carbon (SIC) were calculated using the formula for SOC_i_, in which C_org_ (soil organic carbon) is replaced by TN_i_ (total soil nitrogen) and TIC_i_ (soil inorganic carbon) for each depth (i).

STC_stock_, SIC_stock,_ and STN_stock_ represent the sum of soil total carbon and soil total nitrogen by individual depths of 0–30 cm.

Calculation of soil organic matter (SOM) for each depth (i):

SOM_i_ (%) = Corg_i_ × 1.724 (3)

The C/N ratio was determined by dividing the STC value by the STN value for each depth (i).

### 4.6. Statistical Analyses

Statistical analyses were performed using the Statistical Package for the Social Sciences (IBM SPSS 22.0, Chicago, IL, USA). Linear mixed models were applied to analyze the effect of the studied factors (grassland management, soil depth) on different physical and chemical properties. In the models that used physical (VWC, POR, BD, CF) and chemical soil properties (SOC, SIC, STN, STC, C/N, SOM, pH) as dependent variables, grassland management, soil depth, and their interaction were included as fixed factors, and altitude and precipitation were included as random effects. Significant differences were evaluated at the 0.05 level using a Bonferroni test. The results are reported as estimated fixed-effect means ± standard errors (S.E.s). The Pearson correlation coefficients between studied physicochemical soil properties were calculated for each grassland-management system at a depth of 0–30 cm.

## 5. Conclusions

The results indicate that the management system has a significant influence on soil organic carbon, soil total nitrogen and soil organic matter. In accordance with previous studies, our results show clear differences in soil organic carbon and soil total nitrogen at different depths. Furthermore, we determined that management systems have a notable impact on these soil properties at different depths. A combined system appears to be the optimal choice for reducing carbon and nitrogen losses in the Central European region. It is suggested that less frequent grazing with combined cutting could be the key to sustaining agriculture in the long term while reducing fossil fuel consumption and ensuring a higher level of animal welfare. We believe that longer studies will provide more time for the accumulation of differences in carbon sequestration and thus have a greater impact on higher organic matter storage in different management systems. Further research should be conducted on the long-term monitoring and sampling of grasslands in this region of Europe to expand upon our current knowledge of the role of dominant plant species in above- and belowground processes and to improve our understanding of the mechanisms of grassland ecosystem stability. More in-depth studies are also needed to investigate the combined effects of grazing and cutting on soil nutrients, which will require additional research on this management system. Our findings provide the basis for a better understanding of the effects of grassland management on soil properties, which is important for sustainable agriculture and the environment.

## Figures and Tables

**Table 1:** The effect of management system and soil depth on studied physicochemical properties of soil.

Soil Properties	Management System	Soil Depth
Physical		
VWC	*	***
POR	ns	***
BD	ns	***
CF	ns	**
Chemical		
SOC	ns	***
SIC	ns	ns
STC	ns	***
STN	ns	***
C/N	ns	*
SOM	ns	***
pH	ns	ns

Significance levels are as follows: * *p* ≤ 0.05; ** *p* ≤ 0.01; *** *p* ≤ 0.001; ns—non-significant. SOC—soil organic carbon; SIC—soil inorganic carbon; STC—soil total carbon; STN—soil total nitrogen; SOM—soil organic matter; VWC—volumetric soil water content; BD—bulk density; POR—porosity; CF—mass consisting of coarse fragments; C/N—carbon/nitrogen ratio. The interaction between the management system and soil depth was insignificant in all physical and chemical properties of the soil.

**Table 2:** Soil physical properties under three different grassland-management systems (grazing, cutting and a combination of both) at three depths (0–10, 10–20 and 20–30 cm).

Management System	Soil Depth			*p*-Value
	0–10	10–20	20–30	
VWC (%)				
Grazing	47.49 ± 4.37 a	40.43 ± 3.06 b	36.37 ± 2.80 c	<0.001
Combined	41.99 ± 4.62	35.86 ± 3.32	35.88 ± 3.02	0.404
Cutting	43.87 ± 4.05 a	37.83 ± 2.73 b	35.41 ± 2.51 c	<0.001
*p*-value	0.104	0.203	0.681	
POR (%)				
Grazing	52.46 ± 2.59 a	44.17 ± 2.76 b	39.86 ± 2.17 c	<0.001
Combined	53.27 ± 3.05 a	41.54 ± 3.08 b	37.28 ± 2.57 c	<0.001
Cutting	53.40 ± 2.08 a	42.40 ± 2.39 b	39.06 ± 1.70 c	<0.001
*p*-value	0.151	0.626	0.670	
BD (g cm^−3^)				
Grazing	1.12 ± 0.05 Ac	1.30 ± 0.05 b	1.39 ± 0.05 a	<0.001
Combined	1.09 ± 0.06 ABc	1.36 ± 0.06 b	1.41 ± 0.05 a	<0.001
Cutting	1.01 ± 0.04 Bc	1.34 ± 0.04 b	1.41 ± 0.05 a	<0.001
*p*-value	0.039	0.549	0.894	
CF (%)				
Grazing	11.35 ± 1.16	10.82 ± 1.32	12.99 ± 2.22	0.527
Combined	9.45 ± 1.46	9.92 ± 1.46	12.96 ± 2.47	0.066
Cutting	9.00 ± 0.85	9.68 ± 1.13	11.14 ± 1.93	0.178
*p*-value	0.894	0.720	0.221	

(a, b, c): Different letters indicate significantly different means based on a Bonferroni test with *p* < 0.05 at each depth within each management type. (A, B): Different letters indicate significantly different means based on a Bonferroni test with *p* < 0.05, for each management type within the different depths. Values are represented as estimated fixed-effect means ± standard errors (S.E.s). VWC—volumetric soil water content; BD—bulk density; POR—porosity; CF—mass consisting of coarse fragments.

**Table 3:** Soil chemical properties under three different grassland-management systems (grazing, cutting and combined (a combination of both) system) at three depths (0–10, 10–20 and 20–30 cm).

Management System	Soil Depth			*p*-Value
	0–10	10–20	20–30	
SOC (Mg ha^−1^)				
Grazing	36.34 ± 1.55 a	25.39 ± 2.34 b	19.05 ± 3.40 c	<0.001
Combined	33.62 ± 1.99 a	23.59 ± 2.58 b	20.03 ± 3.65 c	<0.001
Cutting	32.10 ± 1.10 a	25.76 ± 2.08 b	20.49 ± 3.09 c	<0.001
*p*-value	0.073	0.727	0.687	
SIC (Mg ha^−1^)				
Grazing	2.00 ± 1.13	3.35 ± 1.68	4.10 ± 1.68	0.592
Combined	1.93 ± 1.46	2.66 ± 2.17	2.66 ± 2.17	0.951
Cutting	1.49 ± 0.80	2.16 ± 1.19	1.93 ± 1.18	0.895
*p*-value	0.920	0.780	0.487	
STC (Mg ha^−1^)				
Grazing	38.35 ± 1.73 a	28.41 ± 2.89 b	22.72 ± 7.27 c	<0.001
Combined	35.55 ± 2.23 a	25.45 ± 3.24 b	21.35 ± 2.86 c	<0.001
Cutting	33.59 ± 1.22 a	27.59 ± 2.52 b	22.25 ± 1.84 c	<0.001
*p*-value	0.104	0.593	0.859	
STN (Mg ha^−1^)				
Grazing	3.73 ± 0.21 Aa	2.64 ± 0.26 b	1.86 ± 0.26 c	<0.001
Combined	3.55 ± 0.25 Ba	2.56 ± 0.30 b	1.96 ± 0.30 c	<0.001
Cutting	3.21 ± 0.16 Ba	2.65 ± 0.22 b	2.02 ± 0.21 c	<0.001
*p*-value	0.039	0.952	0.766	
C/N				
Grazing	10.35 ± 0.51 b	11.83 ± 1.03 b	16.18 ± 2.05 Aa	0.007
Combined	10.08 ± 0.66	10.38 ± 1.83	11.57 ± 2.64 AB	0.815
Cutting	10.72 ± 0.37	10.99 ± 0.73	11.56 ± 1.45 B	0.819
*p*-value	0.950	0.792	0.016	
SOM (%)				
Grazing	6.40 ± 0.43 a	3.77 ± 0.44 b	2.73 ± 0.40 c	<0.001
Combined	5.89 ± 0.52 a	3.33 ± 0.49 b	2.79 ± 0.46 c	<0.001
Cutting	6.12 ± 0.33 a	3.71 ± 0.39 b	2.84 ± 0.35 c	<0.001
*p*-value	0.560	0.679	0.938	
pH				
Grazing	6.49 ± 0.32	6.56 ± 0.32	6.67 ± 0.32	0.893
Combined	6.41 ± 0.34	6.53 ± 0.35	6.57 ± 0.35	0.950
Cutting	6.30 ± 0.28	6.35 ± 0.28	6.45 ± 0.27	0.869
*p*-value	0.716	0.607	0.639	

(a, b, c) Different letters indicate significantly different means based on a Bonferroni test with *p* < 0.05 at each depth within each management type. (A, B): Different letters indicate significantly different means based on a Bonferroni test with *p* < 0.05, for each management type within the different depths. Values are represented as estimated fixed-effect means ± standard errors (S.E.s). SOC—soil organic carbon; SIC—soil inorganic carbon; STC—soil total carbon; STN—soil total nitrogen; SOM—soil organic matter. C/N—carbon/nitrogen ratio.

## Data Availability

The data may be made available from the authors upon reasonable request.

## Supplementary Materials

The following supporting information can be downloaded at: https://www.mdpi.com/article/10.3390/plants13060838/s1, Figure S1: SOC and SIC concentration under three grassland-management systems (cutting, grazing and combined system) at each depth (0–30 cm).

## References

[1] Poyda A., Reinsch T., Struck I.J., Skinner R.H., Kluß C., Taube F. (2020). Low assimilate partitioning to root biomass is associated with carbon losses at an intensively managed temperate grassland. Plant Soil.

[2] Leifeld J., Fuhrer J. (2009). Long-term management effects on soil organic matter in two cold, high-elevation grasslands: Clues from fractionation and radiocarbon dating. Eur. J. Soil Sci..

[3] Chen L., Baoyin T., Xia F. (2022). Grassland management strategies influence soil C, N, and P sequestration through shifting plant community composition in a semi-arid grasslands of northern China. Ecol. Indic..

[4] Koncz P., Vadász-Besnyői V., Csathó A.I., Nagy J., Szerdahelyi T., Tóth Z., Pintér K., Fóti S., Papp M., Balogh J. (2020). Carbon uptake changed but vegetation composition remained stable during transition from grazing to mowing grassland management. Agric. Ecosyst. Environ..

[5] Lal R. (2004). Soil carbon sequestration to mitigate climate change. Geoderma.

[6] Phukubye K., Mutema M., Buthelezi N., Muchaonyerwa P., Cerri C., Chaplot V. (2022). On the impact of grassland management on soil carbon stocks: A worldwide meta-analysis. Geoderma Reg..

[7] Abdalla M., Hastings A., Chadwick D.R., Jones D.L., Evans C.D., Jones M.B., Rees R.M., Smith P. (2018). Critical review of the impacts of grazing intensity on soil organic carbon storage and other soil quality indicators in extensively managed grasslands. Agric. Ecosyst. Environ..

[8] Chang J., Ciais P., Gasser T., Smith P., Herrero M., Havlik P., Obersteiner M., Guenet B., Goll D.S., Li W. (2021). Climate warming from managed grasslands cancels the cooling effect of carbon sinks in sparsely grazed and natural grasslands. Nat. Commun..

[9] Jones M.B., Donnelly A. (2004). Carbon sequestration in temperate grassland ecosystems and the influence of management, climate and elevated CO_2_. New Phytol..

[10] Wang Z.-P., Han X.-G., Chang S.X., Wang B., Yu Q., Hou L.-Y., Li L.-H. (2013). Soil organic and inorganic carbon contents under various land uses across a transect of continental steppes in Inner Mongolia. Catena.

[11] Dumanski J. (2004). Carbon Sequestration, Soil Conservation, and the Kyoto Protocol: Summary of Implications. Clim. Chang..

[12] Franzluebbers A.J., Conant M.A.R. (2010). Soil organic carbon in managed pastures of the southeastern United States of America. Grassland Carbon Sequestration: Management, Policy and Economics.

[13] Jafarian Z., Kavian A. (2013). Effects of Land-Use Change on Soil Organic Carbon and Nitrogen. Commun. Soil Sci. Plant Anal..

[14] Wu G.-L., Liu Z.-H., Zhang L., Hu T.-M., Chen J.-M. (2010). Effects of Artificial-Grassland Establishment on Plant Community and Soil Properties in a Black-Soil-Type Degraded Grassland. Plant Soil.

[15] Poeplau C. (2021). Grassland soil organic carbon stocks along management intensity and warming gradients. Grass Forage Sci..

[16] Lettens S., Van Orshoven J., van Wesemael B., Muys B. (2004). Soil organic and inorganic carbon contents of landscape units in Belgium derived using data from 1950 to 1970. Soil Use Manag..

[17] Bengtsson J., Bullock J.M., Egoh B., Everson C., Everson T., O’Connor T., O’Farrell P.J., Smith H.G., Lindborg R. (2019). Grasslands—More important for ecosystem services than you might think. Ecosphere.

[18] Ward S.E., Smart S.M., Quirk H., Tallowin J.R., Mortimer S.R., Shiel R.S., Wilby A., Bardgett R.D. (2016). Legacy effects of grassland management on soil carbon to depth. Glob. Chang. Biol..

[19] Eurostat. https://ec.europa.eu/eurostat/data/database.

[20] Chang J., Ciais P., Viovy N., Soussana J.F., Klumpp K., Sultan B. (2017). Future productivity and phenology changes in European grasslands for different warming levels: Implications for grassland management and carbon balance. Carbon Balance Manag..

[21] Crème A., Rumpel C., Malone S.L., Saby N.P.A., Vaudour E., Decau M.-L., Chabbi A. (2020). Monitoring Grassland Management Effects on Soil Organic Carbon—A Matter of Scale. Agronomy.

[22] Wang T., Kang F., Cheng X., Han H., Ji W. (2016). Soil organic carbon and total nitrogen stocks under different land uses in a hilly ecological restoration area of North China. Soil Tillage Res..

[23] Conant R.T., Cerri C.E., Osborne B.B., Paustian K. (2017). Grassland management impacts on soil carbon stocks: A new synthesis. Ecol. Appl..

[24] Rivera J.E., Chará J. (2021). CH_4_ and N_2_O Emissions From Cattle Excreta: A Review of Main Drivers and Mitigation Strategies in Grazing Systems. Front. Sustain. Food Syst..

[25] Clark H., Pinares-Patino C., De Klein C. (2015). Methane and nitrous oxide emissions from grazed grasslands. Grassland: A Global Resource.

[26] Li Z., Liu C., Dong Y., Chang X., Nie X., Liu L., Xiao H., Lu Y., Zeng G. (2017). Response of soil organic carbon and nitrogen stocks to soil erosion and land use types in the Loess hilly–gully region of China. Soil Tillage Res..

[27] Egan G., Crawley M.J., Fornara D.A. (2018). Effects of long-term grassland management on the carbon and nitrogen pools of different soil aggregate fractions. Sci. Total Environ..

[28] Tan W.-F., Zhang R., Cao H., Huang C.-Q., Yang Q.-K., Wang M.-k., Koopal L.K. (2014). Soil inorganic carbon stock under different soil types and land uses on the Loess Plateau region of China. Catena.

[29] Xu T., Zhang M., Ding S., Liu B., Chang Q., Zhao X., Wang Y., Wang J., Wang L. (2021). Grassland degradation with saline-alkaline reduces more soil inorganic carbon than soil organic carbon storage. Ecol. Indic..

[30] Chen L., Wang K., Baoyin T. (2021). Effects of grazing and mowing on vertical distribution of soil nutrients and their stoichiometry (C: N: P) in a semi-arid grassland of North China. Catena.

[31] Guidi C., Vesterdal L., Gianelle D., Rodeghiero M. (2014). Changes in soil organic carbon and nitrogen following forest expansion on grassland in the Southern Alps. For. Ecol. Manag..

[32] Gilmullina A., Rumpel C., Blagodatskaya E., Chabbi A. (2020). Management of grasslands by mowing versus grazing—Impacts on soil organic matter quality and microbial functioning. Appl. Soil Ecol..

[33] Sanaullah M., Chabbi A., Lemaire G., Charrier X., Rumpel C. (2009). How does plant leaf senescence of grassland species influence decomposition kinetics and litter compounds dynamics?. Nutr. Cycl. Agroecosyst..

[34] Senapati N., Chabbi A., Gastal F., Smith P., Mascher N., Loubet B., Cellier P., Naisse C. (2014). Net carbon storage measured in a mowed and grazed temperate sown grassland shows potential for carbon sequestration under grazed system. Carbon Manag..

[35] Tälle M., Deák B., Poschlod P., Valkó O., Westerberg L., Milberg P. (2016). Grazing vs. mowing: A meta-analysis of biodiversity benefits for grassland management. Agric. Ecosyst. Environ..

[36] Sonneveld M.P.W., Van Den Akker J.J.H. (2010). Quantification of C and N stocks in grassland topsoils in a Dutch region dominated by dairy farming. J. Agric. Sci..

[37] Nwaogu C., Okeke O.J., Fashae O., Nwankwoala H. (2018). Soil organic carbon and total nitrogen stocks as affected by different land use in an Ultisol in Imo Watershed, southern Nigeria. Chem. Ecol..

[38] Fynn A.J., Alvarez P., Brown J.R., George M.R., Kustin C., Laca E.A., Oldfield J.T., Schobr T., Neely Wong C.P., Abberton M., Conant R., Batello C. (2010). Soil carbon sequestration in United States rangelands. Grassland Carbon Sequestration: Management, Policy and Economics.

[39] Reeder J.D., Schuman G.E., Morgan J.A., Lecain D.R. (2004). Response of organic and inorganic carbon and nitrogen to long-term grazing of the shortgrass steppe. Environ. Manag..

[40] Wang L., Xu H., Zhang H., Zhang Y. (2022). Grazing and Mowing Affect the Carbon-to-Nitrogen Ratio of Plants by Changing the Soil Available Nitrogen Content and Soil Moisture on the Meadow Steppe, China. Plants.

[41] Wang Z., Guo S., Sun Q., Li N., Jiang J., Wang R., Zhang Y., Liu Q., Wu D., Li R. (2015). Soil organic carbon sequestration potential of artificial and natural vegetation in the hilly regions of Loess Plateau. Ecol. Eng..

[42] Hu Y.-Y., Wei H.-W., Zhang Z.-W., Hou S.-L., Yang J.-J., Wang J.-F., Lü X.-T. (2020). Changes of plant community composition instead of soil nutrient status drive the legacy effects of historical nitrogen deposition on plant community N:P stoichiometry. Plant Soil.

[43] Batjes N.H. (2014). Total carbon and nitrogen in the soils of the world. Eur. J. Soil Sci..

[44] Soussana J.-F., Loiseau P., Vuichard N., Ceschia E., Balesdent J., Chevallier T., Arrouays D. (2004). Carbon cycling and sequestration opportunities in temperate grasslands. Soil Use Manag..

[45] Bardgett R.D., Keiller S., Cook R., Gilburn A.S. (1998). Dynamic interactions between soil animals and microorganisms in upland grassland soils amended with sheep dung: A microcosm experiment. Soil Biol. Biochem..

[46] Laliberté E., Shipley B., Norton D.A., Scott D. (2012). Which plant traits determine abundance under long-term shifts in soil resource availability and grazing intensity?. J. Ecol..

[47] Zhu Y., Delgado-Baquerizo M., Shan D., Yang X., Eldridge D.J. (2021). Grazing impacts on ecosystem functions exceed those from mowing. Plant Soil.

[48] Rousk J., Brookes Philip C., Bååth E. (2009). Contrasting Soil pH Effects on Fungal and Bacterial Growth Suggest Functional Redundancy in Carbon Mineralization. Appl. Environ. Microbiol..

[49] Liptzin D., Norris C.E., Cappellazzi S.B., Bean G.M., Cope M., Greub K.L.H., Rieke E.L., Tracy P.W., Aberle E., Ashworth A. (2022). An evaluation of carbon indicators of soil health in long-term agricultural experiments. Soil Biol. Biochem..

[50] Pringle M.J., Allen D.E., Phelps D.G., Bray S.G., Orton T.G., Dalal R.C. (2014). The effect of pasture utilization rate on stocks of soil organic carbon and total nitrogen in a semi-arid tropical grassland. Agric. Ecosyst. Environ..

[51] Ankenbauer K.J., Loheide S.P. (2017). The effects of soil organic matter on soil water retention and plant water use in a meadow of the Sierra Nevada, CA. Hydrol. Process..

[52] Kemmitt S., Wright D., Goulding K., Jones D. (2006). pH regulation of carbon and nitrogen dynamics in two agricultural soils. Soil Biol. Biochem..

[53] Spohn M., Stendahl J. (2024). Soil carbon and nitrogen contents in forest soils are related to soil texture in interaction with pH and metal cations. Geoderma.

[54] Liu Y., Dang Z.Q., Tian F.P., Wang D., Wu G.L. (2016). Soil Organic Carbon and Inorganic Carbon Accumulation Along a 30-year Grassland Restoration Chronosequence in Semi-arid Regions (China). Land Degrad. Dev..

[55] Hewins D.B., Lyseng M.P., Schoderbek D.F., Alexander M., Willms W.D., Carlyle C.N., Chang S.X., Bork E.W. (2018). Grazing and climate effects on soil organic carbon concentration and particle-size association in northern grasslands. Sci. Rep..

[56] Whitehead D. (2020). Management of Grazed Landscapes to Increase Soil Carbon Stocks in Temperate, Dryland Grasslands. Front. Sustain. Food Syst..

[57] Grčman H., Turniški R., Suhadolc M. (2023). Eutric Cambisols—Slovenia’s best agricultural soils. Geod. Vestn..

[58] Blake G.R. (1965). Particle Density. Methods of Soil Analysis.

[59] (1996). Soil Quality–Determination of Organic and Total Carbon after Dry Combustion.

[60] (1998). Soil Quality–Determination of Total Nitrogen Content by Dry Combustion.

[61] (2005). Soil Quality—Determination of pH.

[62] Šinkovec Marjan B.J., Boštjan M., Helena G., Borut V., Čeh Barbara D.P., Rok M., Denis S., Igor Š. (2021). Soil organic carbon stocks in agricultural land-uses of Slovenia—The multiannual project preliminary report. New Challenges in Agronomy 2021.

