# Supplementary material for: Sustainable Grassland-Management Systems and Their Effects on the Physicochemical Properties of Soil
Source: Plants (Basel). 2024 Mar 14;13(6):838. doi: 10.3390/plants13060838 (PMC10975261; doi:10.3390/plants13060838)
Supplement: Supplementary file 1 [file plants-13-00838-s001.zip › plants-2864615-supplementary.pdf]

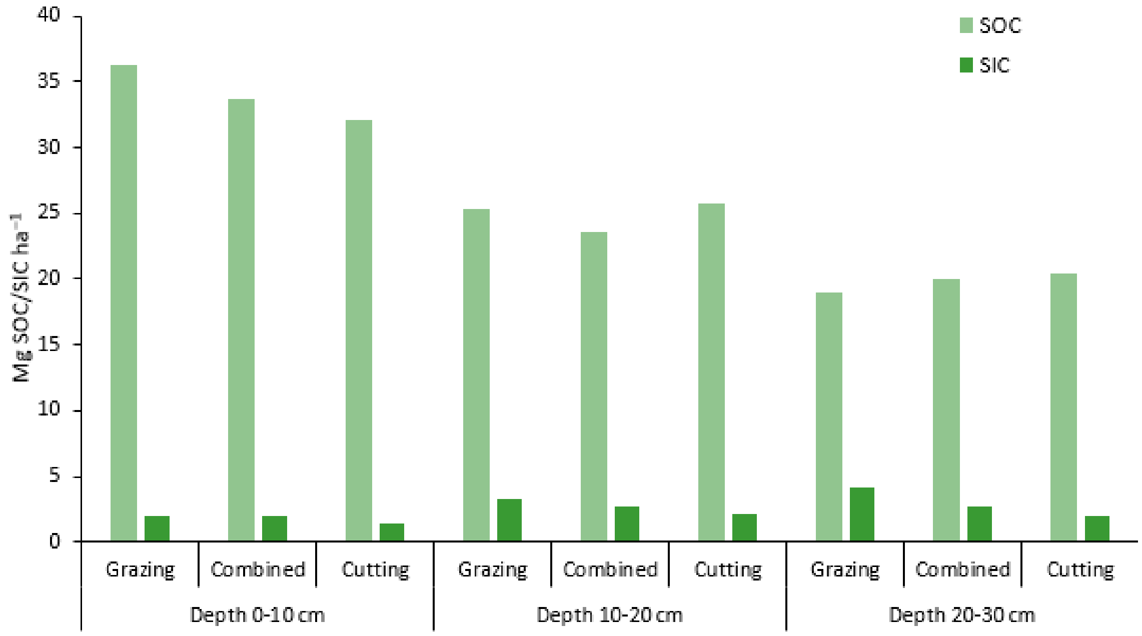

Figure S1: SOC and SIC concentration under three grassland-management systems (cutting, grazing and combined system) at each depth (0–30 cm). SOC—soil organic carbon; SIC—soil inorganic carbon SOC.
